# The Role of Protein Arginine Methylation as a Post-Translational Modification in Cellular Homeostasis and Disease

**DOI:** 10.3390/biology14101370

**Published:** 2025-10-07

**Authors:** Ke Li, Qing Xia, Kexin Li, Wenxin Yan, Changshan Wang

**Affiliations:** 1 School of Life Science, Inner Mongolia University, Hohhot 010020, China; like990614@163.com (K.L.); xiaqing20011230@163.com (Q.X.); kexinli@mail.imu.edu.cn (K.L.); 15065525077@163.com (W.Y.); 2 Inner Mongolia Key Laboratory of Metabolic Disease Regulation and Biological Therapy, Inner Mongolian People’s Hospital, Hohhot 010017, China; 3 Inner Mongolia Academy of Medical Sciences, Inner Mongolian People’s Hospital, Hohhot 010010, China

**Keywords:** protein arginine methyltransferases, cancer, tumor microenvironment

## Abstract

Post-translational modifications (PTMs) of proteins in eukaryotic cells are essential for regulating proteome function and maintaining cellular homeostasis. Among these, the methylation modification of arginine has received much attention in recent years. The enzymatic process of arginine methylation is catalyzed by a family of approximately nine known protein arginine methyltransferases (PRMTs) in humans, which utilize S-adenosylmethionine (SAM) as the methyl group donor. PRMTs are involved in biological processes such as gene transcription, signal transduction, and DNA damage repair. Their role in normal cellular functions and pathological disease states is becoming increasingly clear with the advancement of research. This paper provides a review of the numerous roles of members of the PRMT family in normal cellular function and disease pathophysiology, with a focus on their association with the tumor immune microenvironment (TIME), and discusses their broad impact on various physiological processes and pathological conditions.

## Simple Summary

Post-translational modifications (PTMs) of proteins in eukaryotic cells are essential for regulating proteome function and maintaining cellular homeostasis. Among these, the methylation modification of arginine has received much attention in recent years. The enzymatic process of arginine methylation is catalyzed by a family of approximately nine known protein arginine methyltransferases (PRMTs) in humans, which utilize S-adenosylmethionine (SAM) as the methyl group donor. This paper provides a review of the numerous roles of members of the PRMT family in normal cellular function and disease pathophysiology, with a focus on their association with the tumor microenvironment, and discusses their broad impact on various physiological processes and pathological conditions.

## 1. Introduction

Previous research on methylation as a post-transcriptional modification was primarily focused on lysine residues [1]. Larsen et al. employed mass spectrometry to identify thousands of methylated arginine residues within the cellular proteome, demonstrating the widespread occurrence of arginine methylation [2]. This finding further confirms that protein arginine methylation constitutes one of the key mechanisms of epigenetic regulation in eukaryotic cells. Arginine methylation is catalyzed by nine protein arginine methyltransferases (PRMT1-9) [3]. These enzymes are evolutionarily conserved and have been identified in species ranging from cnidarians to mammals [4], as well as in yeast, which possesses four distinct PRMT homologs [5]. In eukaryotic cells, arginine residues can undergo methylation in three distinct forms on the guanidino nitrogen group: monomethylarginine (MMA), asymmetric dimethylarginine (ADMA), and symmetric dimethylarginine (SDMA) [6,7,8].

**Figure 1:**
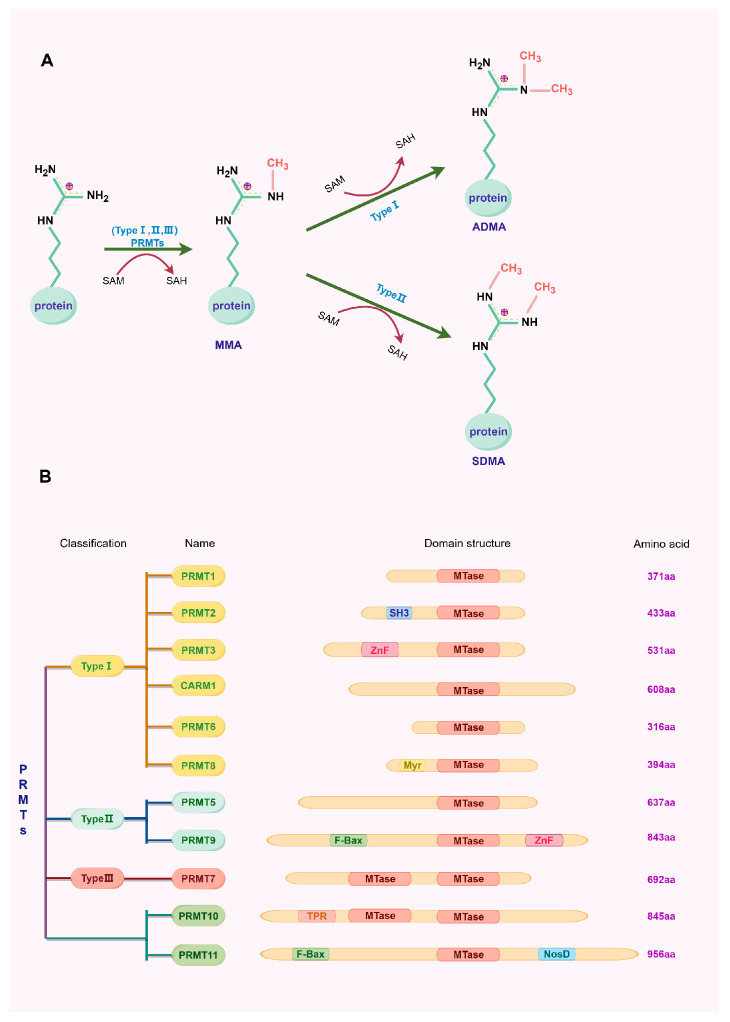
This figure consists of two parts, (**A**) and (**B**). (**A**) illustrates the reaction catalyzed by PRMT isozymes. Enzymes are classified based on the type of reaction they catalyze. PRMTs that generate asymmetrically dimethylated arginine residues (ADMA) are classified as type I PRMTs, while those producing symmetrically dimethylated arginine residues (SDMA) are categorized as type II PRMTs. (**B**) displays the 11 members of the PRMT family. Each member possesses unique structural elements, most notably a conserved MTase domain containing the common post I, II, and III motifs. Accessory domains also include SH3 domains, nitric oxide synthase accessory protein (NosD), F-box domains, tetratricopeptide repeat domains (TPR), myristoylation domains (Myr), and zinc finger domains (<ZnF>). (By Figdraw.).

Based on the methylation marks on the arginine residues, the nine PRMT family members are classified into three classes. PRMT1, PRMT2, PRMT3, PRMT4 (also known as CARM1), PRMT6, and PRMT8 are type I enzymes that catalyze the formation of MMA intermediates by providing a methyl group using s-adenosylmethionine (SAM) and then proceed to a second SAM molecule by adding an additional methyl group to eventually form ADMA. Type II enzymes, comprising PRMT5 and PRMT9, also produce MMA but ultimately catalyze the formation of symmetric dimethylarginine (SDMA). PRMT7 is classified as the sole type III enzyme, as it exclusively generates MMA. PRMT10 and PRMT11, which are bona fide members of the PRMT family but lack known human homologs, have been described in plants. The PRMT family is organized around a highly conserved catalytic core with widely dispersed N- and C-terminal regions [3], which implies that PRMTs have distinct substrate specificity. In histones, the most characteristic methylation of arginine residues includes the R2, R8, R17, and R26 sites of histone H3, as well as the R3 sites of histones H4 and H2A [9]. Moreover, the methylation status of arginine residues equips adjacent chromatin regions with transcriptional activation activity or transcriptional repression activity, which plays a major role in gene regulation (Figure 1).

## 2. Type I Protein Arginine Methyltransferase

Although type I arginine methyltransferase members can catalyze the formation of MMA and ADMA, they primarily function through ADMA, with MMA serving as a transient intermediate. Current evidence indicates that these enzymes are frequently overexpressed in various cancers and generally exhibit oncogenic properties, despite variations in their precise mechanisms and regulatory pathways. The roles and functions of individual type I PRMT members are summarized below.

### 2.1. PRMT1

Protein Arginine Methyltransferase 1 (PRMT1) was the first mammalian protein arginine methyltransferase to be discovered and identified. It is responsible for more than 85% of protein arginine methylation [10]. PRMT1 has broad substrate specificity and can methylate glycine- and arginine-rich residues in the GAR sequences [11]. It can methylate histones and stimulate the transcriptional activity of downstream target genes by methylating histone 4 at the arginine 3 site, generating the H4R3me2a mark. By depositing this mark, PRMT1 stimulates the expression of downstream target genes, thereby influencing diverse cellular physiological processes and fate decisions [12,13,14]. In addition, PRMT1 can also methylate non-histone proteins. For instance, it methylates the DNA repair proteins 53BP1 and MRE11, which help to localize and regulate nucleic acid exonuclease activity, respectively, and are required for the control of DNA damage checkpoints [14,15]. PRMT1 has also been shown to methylate telomeric repeat binding factor 2 (TRF2), which maintains telomere length and protects telomeres from being used as DNA recognized by damage. Consistent with these critical roles, the deletion of PRMT1 leads to telomerase shortening, and knockout of the PRMT1 gene in mice even leads to embryonic lethality [9,16], possibly because PRMT1 plays an important role in the control of cell division coordination [17].

Intensive studies on solid tumors and hematologic malignancies have revealed that PRMT1 is overexpressed or aberrantly spliced in breast, prostate, lung, colon, bladder, and gastric cancers, as well as leukemia [18,19], and its catalytic epigenetic transcriptional activation marker correlates with tumor grade and overall prognosis of solid tumors (Table 1). PRMT1 has also been shown to have a significant role in cell proliferation/viability and cell cycle progression. Depletion of PRMT1 resulted in a significant decrease in the proliferation of osteosarcoma, breast, bladder, and lung cancer cell lines. This reduction in cell proliferation was associated with cell cycle arrest at the G0/G1 phase. Breast cancer cells showed a loss of cyclin D1 and an increase in p21cip1 expression, indicative of cell cycle arrest at this phase. PRMT1 can methylate the estrogen receptor ERα within the DNA-binding domain, thereby regulating the ER signaling pathway to promote breast cancer cell proliferation. A recent study indicates that ERα is methylated specifically by the arginine methyltransferase PRMT1 at arginine 260 in the DNA-binding domain of the receptor. This methylation event is required for mediating the extra-nuclear function of the receptor, enabling its interaction with Src/FAK and p85 and propagating the signal to downstream transduction cascades that orchestrate cell proliferation and survival [20]. Conversely, PRMT1 has also been demonstrated to methylate crucial cytoplasmic proteins that are associated with the apoptotic signaling pathway. It can also act directly on AKT substrates, such as FOXO1 and BCL-2 cell death antagonists (BAD), and directly inhibit AKT phosphorylation on both substrates [21,22]. This methylation prevents PKB/Akt-mediated phosphorylation, thus preventing its inactivation, resulting in enhanced apoptosis. This may seem contradictory, but it actually reveals the high background dependence of PRMT1’s functions. The ultimate effect may depend on cell type and the oncogenic signaling network: in breast cancer that relies on ERα signaling, its proliferation-promoting pathway is dominant. However, it is different in other environments, or it depends on the difference in substrate competition and selectivity. A study demonstrated that PRMT1 was able to induce the EMT process and enhance the capabilities of migration and invasion in breast cancer cells. Meanwhile, the knockdown of PRMT1 not only suppressed metastasis in vivo in mice but also provoked cellular senescence in breast cancer cells. These functional effects of PRMT1 were exerted through the control of ZEB1 transcriptional expression via H4R3me2as modification at the gene’s promoter [23]. Among the substrates of PRMT1, H4R3 is the most popular and of great concern. In prostate cancer research, H4R3diMe was positively correlated with tumor grade progression. The relationship between PRMT1 and H4R3diMe was also investigated in an erythroid cell line, and H4R3 methylation was almost completely inhibited after PRMT1 knockdown. The data showed that the level of the asymmetric dimethylated modification of H4R3 was upregulated in glioma cells, consistent with PRMT1 expression.

PRMT1 is the most extensively studied type I PRMT. It profoundly influences TIME through various mechanisms: it methylates the Arg133 site of cGAS, suppressing its dimerization and activity, thereby blocking the cGAS-STING pathway and downstream interferon responses, which weakens anti-tumor immunity [24]. In gastric cancer, tumor cells utilize this mechanism to inhibit M1 macrophage polarization [25]. Impediment of T Cell Recruitment: In melanoma, PRMT1 upregulates DNMT1 expression by maintaining H4R3me2a and H3K27ac modifications, suppresses endogenous retrovirus (ERV) transcription, and reduces dsRNA accumulation, thereby inhibiting the type I interferon pathway and the production of T cell chemokines (such as CXCL9/10/11), leading to reduced infiltration of CD8^+^ T cells [26]. Mediation of Drug Resistance and Immunosuppression: In triple-negative breast cancer (TNBC), PRMT1 methylates PARP1 (R18), enhancing DNA repair and leading to chemotherapy resistance, and activates the NF-κB pathway to promote the secretion of factors such as IL-1β, maintaining the characteristics of cancer stem cells, reducing CD8^+^ T cell infiltration, and inducing the expression of inhibitory immune checkpoints like LAG3, thereby creating an immunosuppressive microenvironment [27].

In summary, PRMT1 is not merely a simple “oncoprotein,” but rather a multifunctional hub for cellular state regulation. Its role in cancer is context-dependent, driving tumor progression through both intracellular effects (regulating proliferation, apoptosis, and DNA repair) and extracellular effects (shaping the immunosuppressive TME).

**Table 1:** Role of protein arginine methyltransferases 1 in cancer pathogenesis, anti-tumor immunity, drug resistance, and cancer immunotherapy.

Cancer Type	Role(s) in Cancer	Mechanism of Action	Ref.
Acute myelogenous leukemia	Cell transformation	PRMT1 + KDM4C→epigenetic reprogramming↑→AML cell transformation↑	[28]
	Cell survival and proliferation	PRMT1→methylation of FLT3→survival and proliferation of FLT3-ITD AML cell↑	[29]
Breast cancer	Cell proliferation/viability and cell cycle progression	PRMT1→methylation of ERα→interacts with Src/FAK and p85→cell proliferation and survival	
		PRMT1→EZH2 methylation→inhibits P16 and P21 transcriptional expression→promotes cell cycle progression	[30]
Colorectal cancer	Promotion of glycolysis, proliferation, and tumorigenesis	PRMT1→meR206-PGK1→pS203-PGK1↑→glycolytic activity and CRC tumorigenesis	[31]
	Cell proliferative, colony-formative, and migratory abilities	PRMT1→H4R3me2a→recruits SMARCA4→EGFR signaling↑→proliferative, colony-formative, and migratory abilities↑	[32]
Gastric cancer	Cell migration and metastasis	PRMT1→ recruits MLXIP→β-catenin transcription and the β-catenin signaling pathway↑→GC cell migration and metastasis	[33]
Hepatocellular carcinoma	Cell proliferation and xenograft tumor growth	CDK5→phosphorylation of PRMT1→methylation of WDR24→mTORC1 pathway↑	[34]
Human melanoma	Tumor growth and metastasis	PRMT1→methylation of ALCAM→tumor growth and metastasis	[35]
Lung cancer	DNA repair ability and chemotherapeutic drug resistance	PRMT1→methylation of FEN1→DNA repair ability and drug resistance	[36]
	Cell invasion and drug resistance	PRMT1→methylation of Twist1 and p120-catenin expression↑→transcription of Kaiso↑→EMT in Osimertinib-resistant cells↑	[37]
Multiple myeloma	Cell proliferation and apoptosis	PRMT1→methylation of WTAP→NDUFS6 m6A modification →MM cell proliferation and OCR levels ↑ cell apoptosis and ROS levels↓	[38]
Ovarian cancer	Cell migration and invasion	PRMT1→Methylation of BRD4→phosphorylation of BRD4→migration and invasion	[39]
Pancreatic cancer	Apoptosis	stress response →PRMT1→methylation of p14ARF→p53—independent apoptosis	[40]
Prostate cancer	PCa cell growth	PRMT1 + circ_0094606→methylation of ILF3→stability of IL-8 mRNA↑→M2 polarization of macrophages→PCa growth↑	[41]

Arrows indicate direction of effect: (→) leads to/causes; (↑) increase/upregulation/activation; (↓) decrease/downregulation/inhibition.

### 2.2. PRMT2

Protein Arginine Methyltransferase 2 (PRMT2) contains a methyltransferase domain and an SH3 domain. Initially, it was not thought to have methyltransferase activity, but subsequent studies have identified very weak type I enzyme activity from this enzyme and demonstrated its function as a coactivator of androgen and estrogen receptors [42]. This activation activity appears to be dependent on the integrity of the methyltransferase structural domain [43,44]. Zhong et al. reported the molecular and cellular biology of four novel PRMT2 splice variants isolated from breast cancer cells, referred to as PRMT2L2, PRMT2α, PRMT2β, and PRMT2γ. However, the relative expression levels and specific functional roles of these isoforms remain largely unexplored. Evidence has shown that PRMT2 is recruited by β-catenin to histone H3, where it deposits an asymmetric dimethyl mark on R8 of target gene promoters. PRMT2 can hinder nuclear factor kappa B (NF-κB)-dependent transcription, similar to PRMT4, by reducing the interaction between NF-κB and DNA. This leads to an increased susceptibility of cells to apoptosis by inducing the nuclear accumulation of IκB-α. Although mice deficient in PRMT2 are viable and capable of living, studies indicate that PRMT2 induces STAT3 methylation at residue Arg31 in the hypothalamus via the AdoMet binding domain, resulting in reduced swallowing ability and thin stature [45]. A study by Maryem et al. demonstrated that, in primary bone marrow-derived macrophages (BMDMs) [46], the expression of PRMT2 was reduced under high-glucose conditions and inhibited the LXR-mediated expression of ATP-binding cassette transporter protein A1 (ABCA1). In contrast, in a recent study, it was found that the macrophage overexpression of PRMT2 inhibited ox-LDL-induced foam cell formation [47], which may be associated with increased ABCA1 expression and ABCA1-mediated cholesterol efflux. PRMT2 expression was reduced in macrophages from hyperglycemic and diabetic patients, and Beyza et al. discovered that this was causally linked to an increased inflammatory response via macrophages with atherosclerotic-impaired atherosclerosis degeneration [48]. Furthermore, PRMT2 was found to attenuate traf6-mediated antiviral responses, revealing its novel function in the negative regulation of innate immunity [49].

PRMT2 exhibits strikingly divergent functions across different cancer types and even within different models of the same cancer. In glioblastoma, breast cancer, and hepatocellular carcinoma (HCC) [50], PRMT2 acts as a putative oncogene, with its high expression promoting cell proliferation and inhibiting apoptosis through catalyzing H3R8me2a (such as activating Bcl2 expression). However, in cardiac cancer tumors, its downregulated expression suggests potential tumor-suppressive activity [51]. This strong context dependency indicates that the ultimate functional output of PRMT2 heavily relies on its cellular environment, interacting protein networks, and specific pathological contexts. Simply classifying it as an oncogene or tumor suppressor gene is one-sided, and future research must clarify the specific molecular switches underlying its functional transitions. PRMT2 possesses only very weak type I methyltransferase activity. This compels us to critically examine its biological effects, i.e., whether its oncogenic role relies on its weak catalytic activity or its function as a protein interaction scaffold. For instance, its roles as a nuclear receptor coactivator and an inhibitor of NF-κB may depend more on its physical association with other transcriptional complexes rather than its catalytic activity. Many studies attribute its effects to H3R8me2a, but given its extremely weak enzymatic activity, these histone modification marks likely require the cooperation of other highly active PRMTs, such as PRMT1 or PRMT6, or its critical substrates might be certain non-histone proteins.

PRMT2’s discovery in acute myeloid leukemia (AML) is of critical importance, as it reveals the role of PRMT2 beyond the cancer cells themselves. The absence of PRMT2 leads to excessive production of IL-6 and persistent activation of STAT3 by lifting the inhibition on NF-κB signaling, creating a highly inflammatory bone marrow microenvironment. This indicates that PRMT2 is a key nexus between the intrinsic functions of cancer cells and the signaling network of the microenvironment. PRMT2 has been found to play a “dual role” in colorectal cancer (CRC): it intrinsically promotes tumor stemness and metastasis by activating the WNT5A/β-catenin signaling pathway and extrinsically regulates the TIME. PRMT2 can induce the polarization of tumor-associated macrophages (TAMs) towards the M2 phenotype (tumor-promoting, immunosuppressive) and inhibit the function of CD4+ and CD8+ T cells, leading to their exhaustion, thereby synergistically driving tumor progression and immune escape [52].

PRMT2 is a functionally complex and highly context-dependent protein whose role oscillates between “oncogene” and “tumor suppressor”. Its weak enzymatic activity suggests that its scaffolding function may be more critical, and its discovery of mediating inflammation in AML extends its function from cancer cells themselves to the tumor microenvironment, particularly in immune and metabolic regulation.

### 2.3. PRMT3

Protein Arginine Methyltransferase 3 (PRMT3) has a substrate recognition module with a zinc finger structural domain at its N-terminal end and is involved in the regulation of ribosome function and protein synthesis [53]. Ribosomal protein S2 (RpS2) was the first reported substrate for PRMT3 in the cytoplasm, followed by several other PRMT3 substrates reported, such as nuclear poly(A) binding protein (PABPN1), heterogeneous nuclear ribonucleoprotein A1 (hnRNPA1), and glyceraldehyde--3--phosphate dehydrogenase (GAPDH) [54]. The methylation level of RpS2 decreased in cells lacking PRMT3, and the accumulation level of free ribosome subunits decreased in 60S, resulting in the proportion of free ribosome subunits being out of balance, but rRNA precursor processing seemed to proceed normally, allowing PRMT3-deficient cells to survive [55,56]. This was also confirmed in live mouse experiments, where mouse embryos targeted to disrupt PRMT3 were smaller but survived after birth and reached normal mouse size as adults; in these mice, RpS2 was hypermethylated [57]. Also, PRMT3 in yeast methylates RpS2 [55], emphasizing the conserved nature of enzyme–substrate pairing. PRMT3 may also play a key role in neuronal translation through its interaction with RpS2, regulating the maturation of rat dendritic spines [58].

There is a potential role for PRMT3 in the control of tumor growth and disease development. The regulation of PRMT3 may play a role in tumor growth. Studies have shown that the differentially expressed tumor suppressor DAL-1 (differentially expressed in adenocarcinoma of the lung)/4.1B interacts with PRMT3 and inhibits its ability to methylate substrates, affecting the development and progression of lung and breast adenocarcinomas, suggesting that PRMT3 may be an effective inhibitory target for the treatment of cancer [59,60]. Zhang et al. found that methylation of HIF-1α R282 catalyzed by PRMT3 led to reduced polyubiquitination of HIF-1α. In animal models, PRMT3-mediated tumorigenesis was shown to be HIF-1α methylation-dependent. Another study reported the regulation of the key oncogene c-MYC by PRMT3; Hu et al. revealed that PRMT3 inhibits the polyubiquitination of c-MYC, leading to increased stability of c-MYC in CRC cells, thereby promoting the c-MYC-dependent proliferation of CRC cells in vitro and in vivo [61]. It has previously been suggested that PRMT3 promotes cancer progression by reprogramming cancer cell metabolism. Expression of PRMT3 is upregulated in pancreatic cancer and mediates metabolic reprogramming and cell proliferation by causing R248 methylation of GAPDH [54]. In addition, Yu Lei et al. showed that PRMT3 interacts with and mediates the ADMA modification of lactate dehydrogenase A (LDHA) at arginine 112 (R112), so PRMT3 promotes glycolysis and hepatocellular carcinoma growth by enhancing the arginine methylation of LDHA [62]. The aforementioned reports on PRMT3 indicate its involvement in multiple unrelated carcinogenic processes. However, this “multifunctionality” raises a central question: Does PRMT3 possess a unified upstream signaling pathway or a core carcinogenic mechanism? Current research appears fragmented and lacks a main thread.

Recent research has discovered that PRMT3 plays a crucial role in immune evasion in liver cancer. PRMT3 can methylate the R446 site of heat shock protein 60 (HSP60), maintaining its polymerization and normal function. Once PRMT3 is inhibited, the methylation and polymerization of HSP60 are impaired, leading to the disruption of mitochondrial homeostasis and the release of mitochondrial DNA (mtDNA). The cytosolic mtDNA then activates the cGAS/STING signaling pathway, triggering a type I interferon response, which enhances CD8+ T cell infiltration and anti-tumor immunity and increases sensitivity to PD-1 inhibitors. It is noteworthy that PD-1 antibody therapy in liver cancer can upregulate PRMT3 expression through the IFNγ-STAT1 pathway, forming a negative feedback loop that promotes immune resistance. PRMT3 plays the role of a “cytoplasmic oncogenic hub” through various mechanisms (stabilizing oncoproteins and driving metabolic reprogramming), but the complexity and context dependency of its functions necessitate more precise future studies [63].

Furthermore, PRMT3 plays a relatively complex role in the tumor immune microenvironment, primarily promoting immune suppression by regulating mitochondrial homeostasis and innate immune signaling pathways, thereby helping tumor cells evade attacks from the immune system.

### 2.4. PRMT4 (CARM1)

Protein Arginine Methyltransferase 4 (PRMT4), commonly referred to as coactivator-associated arginine methyltransferase 1 (CARM1), was originally identified through its binding to GRIP1, the p160 steroid receptor coactivator. CARM1 exists as a dimer and, similar to other PRMTs, may require dimerization for its methyltransferase activity. Within its catalytic domain, the dimer arm of CARM1 features a unique insertion of 9–10 residues, which results in a central cavity that is larger and less negatively charged than that of the PRMT1 dimer. PRMT4 is involved in thymocyte differentiation, as shown by the lack of thymocyte LAMP-regulated phosphoprotein methylation in Carm1 (−/−) cells. Furthermore, deletion of PRMT4 in mice increases lung cell proliferation and alveolar cell differentiation. Another study found that CARM1 plays a role in the early developmental stages of the mammalian embryo and extraembryonic lineage establishment [64], while inhibition of CARM1 at the blastocyst stage reduces the levels of H3R26me2s, promotes apoptosis, and affects normal blastocyst development [65], indicating that CARM1 is an important protein in the regulation of blastocyst development. In contrast, CARM1 plays the role of a transcriptional coactivator during retinoic acid-induced embryonic stem cell differentiation. It has also been shown that CARM1 can directly methylate the histone acetyltransferase CREB-binding protein (CBP) and p300, which are involved in steroid hormone-dependent transcriptional activation and spermatogenesis [66]. In summary, an increasing number of studies have demonstrated that PRMT4 plays a crucial role in the regulation of various cellular processes, including mRNA processing, tissue development, and the regulation of protein stability. PRMT4 methylates histones and a variety of non-histone substrates, functioning as a coactivator for nuclear hormone receptors and various transcription factors, such as p53, NF-κB, and E2F1.

The most prominent feature of PRMT4 is its highly context-dependent functionality. In breast cancer, prostate cancer, lung cancer [67], and lymphoma [68], it acts as a definitive oncogene by activating oncogenic transcriptional programs (such as c-Myc) through the methylation of histones (H3R17/R26) and chromatin remodelers (e.g., BAF155). For instance, in breast cancer cells, CARM1 binding to enhancers can enhance estrogen receptor-dependent transcriptional activation, thus promoting breast cancer progression [69]. Recently, Wang et al. identified another factor contributing to PRMT4-induced pathogenicity during breast cancer progression and metastasis: protein arginine methylation on R155 of BAF1064, a component of the SWI/SNF chromatin remodeling complex. Methylation of BAF41 positively regulates the expression of the c-Myc pathway, and CARM1/PRMT4 facilitates breast cancer metastasis by methylating the arginine residue R155 of the chromatin remodeling factor BAF1064. However, in hepatocellular carcinoma (HCC) and pancreatic cancer, it exhibits tumor-suppressive properties, such as inhibiting metabolic reprogramming through the methylation of GAPDH. In hepatocellular carcinoma cells, CARM1-mediated GAPDH methylation is a key regulatory mechanism of glucose metabolism [70], retarding the proliferative capacity of cancer cells by regulating the metabolic reprogramming of tumor cells. These observations suggest that the function of CARM1 in cancer is context-dependent. Indeed, CARM1 itself is not inherently defined as “oncogenic” or “tumor-suppressive.” It is a powerful tool for epigenetic and transcriptional regulation. Its ultimate function is dictated by the specific signaling pathway to which it is recruited. When recruited by potent pro-oncogenic transcription factors within active pathways such as ER, Wnt, or NF-κB (e.g., in breast or colorectal cancer), CARM1 acts as an oncogene. Conversely, within the active ASK1-p38/JNK apoptosis pathway (e.g., in the liver), where CARM1 is utilized to promote the clearance of damaged cells, it functions as a tumor suppressor gene.

PRMT4 is not only an intrinsic driver of cancer cells but also an active shaper of the TME. Although direct literature reports are still relatively limited, based on its biological functions within cancer cells and its impact on immune-related factors, we can speculate and summarize its potential mechanisms of action. Firstly, it regulates pro-cancer signaling pathways: activating AKT/mTOR (e.g., hepatocellular carcinoma) [71], Wnt/β-catenin, and other pathways to promote tumor progression, indirectly forming an immunosuppressive microenvironment. Another mechanism is epigenetic regulation: PRMT4 catalyzes the asymmetric dimethylation of histones (e.g., H3R17, H3R26) and non-histone proteins, broadly regulating gene transcription and potentially affecting the expression of immune-related molecules [69]. Lastly, PRMT4 is involved in potential immune modulation: it may directly or indirectly influence immune cell functions (e.g., T cells, macrophages) through methylation modifications, and type I PRMT inhibitors exhibit synergistic effects with immunotherapy [72].

In general, PRMT4 plays a crucial role in regulating various significant factors and pathways. It exhibits diverse functions across different tumors, as well as among distinct subtypes of the same tumor. Additionally, numerous new PRMT4 substrates continue to be identified, but clinically applicable cancer treatment strategies based on CARM1 remain to be explored.

### 2.5. PRMT6

Protein Arginine Methyltransferase 6 (PRMT6) is predominantly localized in the nucleus and, like PRMT1, methylates glycine- and arginine-rich (GAR) motifs [73]. It serves as the primary enzyme responsible for histone H3 arginine 2 (H3R2) methylation in mammalian cells [74]. This modification antagonizes the activating mark H3K4me3, thereby contributing to transcriptional repression. PRMT6 also cooperates with the transcription factor LEF1 to activate the expression of cyclin D1, thereby influencing cell differentiation and proliferation [75]. Additionally, PRMT6 interacts with nuclear receptors [76], potentially through its ability to methylate arginine 3 on the N-terminal tails of histone H4 and H2A [74]. Thus, while PRMT6 can repress transcription via H3R2 methylation, it may also activate transcription through H4R3 or H2AR3 methylation—a hypothesis that remains to be fully validated. Recent studies further demonstrate that PRMT6-mediated H3R2me2a recruits the chromosomal passenger complex (CPC) to chromosome arms, facilitating chromosome condensation [77]. Moreover, PRMT6 inhibits the PI3K–AKT signaling pathway and regulates pre-mRNA splicing by catalyzing the arginine methylation of the phosphatase PTEN [78].

PRMT6 is aberrantly overexpressed in multiple human cancers—including lung [79], liver [80], colon [81], gastric [82], endometrial [47,83], and prostate cancer—and its dysregulation is associated with aberrant methylation patterns and poor clinical prognosis. The oncogenic mechanisms of PRMT6 vary across cancer types. In endometrial carcinoma (EMC), studies conducted both in vitro and in vivo have revealed elevated PRMT6 expression at the mRNA and protein levels. PRMT6 promotes EMC cell migration and proliferation by activating the AKT/mTOR signaling pathway [83], thereby contributing to tumor progression [83]. Cell cycle dysregulation, a hallmark of cancer, often involves aberrant control of cell cycle regulatory proteins. In lung adenocarcinoma (LUAD), protein arginine methylation has been implicated in cell cycle disruption. PRMT6 acts as an oncogene in LUAD by epigenetically repressing p18 expression, which impedes the G1/S phase transition and facilitates tumor cell proliferation in both in vitro and in vivo models [79]. Additionally, PRMT6 has been shown to have tumor-promoting properties in different systems, including the blood system (leukemia), the brain system (glioblastoma), etc. However, PRMT6 regulates aerobic glycolytic responses in hepatocellular carcinoma [84], induces autophagic responses in the malignant microenvironment of hepatocellular carcinoma [85], and affects hepatocarcinogenesis and progression by regulating RAS/RAF binding and MEK/ERK signaling pathways [80]. The aforementioned pathways encompass nearly all core oncogenic mechanisms. Is PRMT6 truly such a “versatile” driver? Or have current studies failed to isolate its most specific and crucial oncogenic functions? This necessitates that future research employ more precise substrate identification and functional complementation assays to differentiate its various roles. Moreover, like PRMT3, PRMT6 affects the antiviral capacity of the organism. On the one hand, PRMT6 reduces binding and export to the virus; on the other hand, it attenuates antiviral innate immunity [86] by blocking the TBK1-IRF3 signaling pathway [87] and decreasing the level of IRF3 phosphorylation [88]. Thus, it affects the organism. However, the mechanisms regulating the stability of the PRMT6 protein in cells remain largely unknown. According to recent research, cellular senescence is essential for inhibiting oncogene-induced carcinogenesis in vivo. The findings point to a potential role for arginine methylation in controlling p53 mRNA during carcinogenesis. Both replicative and DNA damage-induced senescence are associated with a significant drop in PRMT6 protein levels, which is correlated with the induction of the p21 protein and raises the possibility that PRMT6 is involved in the production of p53 target genes. Because increased PRMT6 expression lowers p53 levels and promotes transformation, it may aid in the development of tumors.

Recent studies have revealed that PRMT6 is not only an epigenetic modification enzyme but also a key driver of tumor immune evasion, influencing the tumor immune microenvironment (TIME) through various mechanisms. In hepatocellular carcinoma (HCC), PRMT6 expression is negatively correlated with autophagic flux. It promotes the degradation of its chaperone protein HSC70 by methylating BAG5, thereby inhibiting autophagy. The absence of PRMT6 can induce autophagy, enhancing the survival ability of HCC cells in stress microenvironments and tumorigenesis. This indicates that PRMT6 affects tumor cells’ adaptation and survival to microenvironmental pressures by regulating the methylation of autophagy-related proteins [85]. Promotion of immune evasion characteristics in tumor cells: PRMT6 can facilitate the epithelial-to-mesenchymal transition (EMT), invasion, and metastasis through various mechanisms [89]. These more aggressive tumor cells are generally more effective in resisting attacks from immune cells, indirectly facilitating the formation of an immunosuppressive microenvironment. Potential regulation of immune cell function (requires further investigation): Although direct evidence is limited, PRMT6 is also expressed in immune cells, such as T cells. It is hypothesized that PRMT6 may directly regulate the function, differentiation, or exhaustion of T cells through methylation modification, thereby influencing the intensity of anti-tumor immune responses. However, this still requires more research to confirm.

In summary, PRMT6 belongs to the type I PRMT family and functions by methylating histone or non-histone proteins to control gene expression, stimulate or inhibit signal transduction, promote tumor stem cell self-renewal and differentiation, and promote cancer cell proliferation and migration. The different proteins that PRMT6 methylates in cells are consistent with the different biological processes connected to these functions of PRMT6. Furthermore, PRMT6 inhibitors are anticipated to become new tumor therapeutic targets because of their synergistic effects on tumor cells and components of the tumor microenvironment, which may make them particularly successful in cancer therapy.

### 2.6. PRMT8

Protein Arginine Methyltransferase 8 (PRMT8) shares a high degree of sequence identity with PRMT1 [90], which has a unique N-terminal, two proline-rich sequences that bind many SH3 domains critical for plasma membrane targeting [91]. PRMT8 recombinase has low in vitro activity; however, removal of the N-terminal structural domain by protein hydrolysis or inhibition of s-adenosylmethionine sensitivity leads to increased activity [91,92]. In addition to this, PRMT8 is characterized by tissue-specific expression, mainly on neurons in the brain. Therefore, most studies on PRMT8 have focused on neural networks. As a unique brain-specific protein, PRMT8 can affect neuromotor activity in the organism [93]. It was found that PRMT8 is mainly involved in the somatosensory and limbic systems and is part of the motor system [94]. It has been noted that PRMT8 knockout mice exhibit abnormal motor behavior and reduced visual acuity [95,96], which is because the development of vertebrate neurons requires alterations in cell membrane phosphatidylcholine (PC) metabolism, and PRMT8 can directly hydrolyze PC to produce choline and phosphatidic acid, thus affecting mouse motor behavior. Moreover, deletion of PRMT8 alters synaptic composition and function, leading to behavioral deficits and also exhibiting hippocampus-dependent memory reduction [97], suggesting that PRMT8 can act as an epigenetic regulator of developmental neuroplasticity. In addition, PRMT8 is an important regulatory component of membrane phospholipid composition, short-term memory function, mitochondrial function, and neuroinflammation in response to hypoxic stress [98], and PRMT8 also protects against increased cellular stress due to aging [99], which is required for neuroprotection. In contrast, PRMT8-regulated synaptic actin dynamics are an important regulatory mechanism of the actin cytoskeleton during synapse development [100], which facilitates the maintenance of the structural integrity of synaptic connections within neuronal networks. In addition to neurological studies, PRMT8 also affects stem cell function and can control the pluripotency and mesodermal differentiation of human embryonic stem cells by enhancing the PI3K/AKT/SOX2 pathway through basic fibroblast growth factor (bFGF) [101]. Due to its brain-restricted expression, myristoylation-based membrane anchoring, and dual enzymatic activity of phospholipase and methyltransferase, PRMT8 is distinct from all other PRMTs. Numerous neuronal functions, such as dendritic arborization, dendritic spine maturation, synaptic plasticity, motor function, and visual acuity, have recently been shown to be impacted by PRMT8. Many membrane and cytoplasmic proteins in the brain are arginine-methylated by mass spectrometry.

Numerous cancer forms have been found to have high levels of PRMT8 expression. This is noteworthy because, in mature organisms, PRMT8 prefers to be located in brain tissue, indicating a potential function for dysregulation of PRMT8 in carcinogenesis or the maintenance of cancer cell phenotypes. PRMT8 may prevent the features of colon cancer stem cells from developing. Because PRMT8 is highly expressed in colon cancer stem cells, it may be able to influence the expression of Oct4 and Nanog multipotent transcription factors by preventing Sox2 degradation and raising Sox2 protein content through Sox2 methylation [102]. Therefore, it is considered a potential therapeutic target for the treatment of malignant colon tumors. Among them, high PRMT8 expression was associated with increased patient survival in patients suffering from breast and ovarian cancer, while the opposite was true in gastric cancer, where high PRMT8 expression was associated with decreased patient survival [103]. Furthermore, it has been reported that PRMT8 is almost completely absent in human glioblastoma tissues and may be associated with the development of malignancies in the human brain [104]. Thus, the tissue-specific expression and unique enzymatic activity of PRMT8 allow it to be used as a target for drug development to delay the onset of neurodegenerative diseases and tumor treatment.

PRMT8 may alter immune recognition and escape by regulating the expression of tumor cell surface molecules, such as PD-L1. Upregulating PD-L1 can promote immune escape, while downregulating it may enhance immune killing [105]. PRMT8 may also affect the inflammatory state in the immune microenvironment by modulating inflammation-related signaling pathways, thereby regulating immune cell function and tumor progression.

According to available reports, certain enzymes have been found to interact; for example, PRMT1 and PRMT6 both methylate the GAR motif and similarly methylate H4R3 to activate transcription; CARM1 and PRMT8 play a role in embryonic stem cell differentiation; PRMT3 and PRMT6 play a role in antiviral mechanisms; PRMT1 and PRMT8 play important roles in embryonic and neural development without overlapping, depending on their specific N terminal [106], so in some aspects, they may act synergistically, or there may be complementary relationships that need to be further explored jointly.

## 3. Type II Protein Arginine Methyltransferase

Type II arginine methyltransferases catalyze the formation of MMA and SDMA. Like type I enzymes, MMA functions as an intermediate and eventually forms SDMA; it is also highly expressed in cancer, promoting cancer development. The roles and functions of type II enzyme members are summarized separately below.

### 3.1. PRMT5

Protein Arginine Methyltransferase 5 (PRMT5) consists of four PRMT5 proteins and four essential cofactors, methyl glycosylated protein 50 (MEP50) and WD repeat domain 77 (WDR77), forming a unique hetero-octameric complex with higher methylation activity [107,108]. PRMT5, the major type II arginine methyltransferase, catalyzes the methylation of four arginine residues (H4R3, H2AR3, H3R8, and H3R2) in the histone tail [108], where PRMT5-regulated H4R3me2s are associated with repression of gene expression through histone ubiquitination or DNA methylation [109]. In contrast, PRMT5-catalyzed H3R2me2s bind the WDR5 and MLL family coactivator complexes together, resulting in the formation of H3K4me3 at euchromatin loci and the transcriptional activation of redox-related genes [110]. Thus, unlike PRMT6—which typically represses transcription via H3R2me2a—PRMT5 can activate transcription through H3R2me2s, while its methylation of H4R3 is associated with transcriptional repression. It has been shown that knockout PRMT5 mice show embryonic lethality [111], indicating its role in development. In contrast, in the neural, muscular, hematopoietic, and reproductive systems, PRMT5 mainly maintains tissue homeostasis, as well as stem cell survival and self-renewal capacity [112]. In addition, PRMT5 can protect cellular redox homeostasis by regulating activating transcription factor 4 (ATF4) [113]. On the other hand, the PRMT5 methylation of the cell cycle-associated nuclear transcription factor E2F-1 increases cell viability, while methylated E2F-1 can bind to cyclin A and inhibit PRMT1-induced apoptosis [114,115]. Therefore, PRMT5 and PRMT1 have opposite roles in cell viability, and it remains to be further explored whether other associations exist. Research has demonstrated that PRMT5 regulates the growth of T cells, B cells, and iNKT cells, among other immune cells. For instance, B-cell death and decreased germinal center development are caused by PRMT5 deletion, which is exclusive to B cells [116]. By increasing P53 expression and lowering AKT pathway activity, PRMT5 inhibition induces CD8+ T cell death [117].

PRMT5 overexpression has been reported in many malignancies, including glioblastoma [118], melanoma [119], ovary [120], lung [121], breast [122], stomach [123], and lymphoma [124], which correlates with its ability to directly methylate several transcription factors (Table 2). The mechanisms of PRMT5 in cancer are complex and diverse. Based on its molecular functions and the oncogenic processes it influences, these mechanisms can be categorized as follows: PRMT5 directly silences the expression of key tumor suppressor genes by catalyzing the symmetric dimethylation of histones (e.g., H4R3me2s) or collaborating with non-histone modifying enzymes. Accumulating evidence suggests that PRMT5 may function as an oncogene to drive cancer cell growth by epigenetic inactivation of several tumor suppressors. PRMT5 epigenetically silences tumor suppressor FBW7 expression, leading to elevated cMyc levels and subsequently enhancing proliferation and aerobic glycolysis in pancreatic cancer cells [125]. PRMT5 interacts with c-Myc and inhibits PTEN, CDKN2C (p18INK4C), CDKN1A (p21CIP1 or WAF1), CDKN1C (p57KIP2), and p63 gene expression to promote gastric cancer cell growth [126].

PRMT5 drives cell proliferation by directly affecting the expression or function of cell cycle regulatory proteins. In differentiated versus undifferentiated cells, PRMT5 plays a different role. Furthermore, PRMT5 stimulates cyclin D1 and E1 via ERK signaling while suppressing BTG2 expression to encourage the growth of hepatocellular carcinoma cells.

PRMT5 alters the metabolic patterns of cancer cells by methylating metabolism-related proteins to meet their bioenergetic and biosynthetic demands for rapid growth. PRMT5 may potentially target nonhistone substrates in cancer. For instance, symmetric dimethylation of R321 on sterol regulatory element-binding protein 1 (SREBP1) triggers carcinogenesis and de novo lipogenesis in hepatocellular carcinoma. In addition, it binds to the proximal promoter region of the AR gene; it mainly contributes to the enriched symmetric dimethylation of H4R3 in the same region in prostate cancer and regulates prostate cancer malignancy.

PRMT5 collaborates with known key oncogenic drivers to amplify their signals and jointly promote tumorigenesis. In several lymphomas, PRMT5 acts synergistically with key tumorigenic drivers, including cyclin D1, c-Myc, and NOTCH1, to regulate the lymphomagenic process. A prior study demonstrated that PRMT5 downregulation prevented the G1 phase from repeating and that PRMT5 activity was raised as a result of cyclin D1-CDK4 phosphorylating the molecular chaperone protein MEP50. This in turn prevented the expression of several tumor suppressor genes, which in turn prevented excessive cell proliferation and malignant transformation. These findings suggest that PRMT5 is a critical upstream mediator for cancer cell proliferation and that it regulates the progression of the cell cycle, which is necessary for cell proliferation. This is also associated with miRNA regulation, and it has been demonstrated that PRMT5 promotes lymphoma by increasing cyclin D1 and c-Myc expression through inhibition of miR-33b, miR-96, and miR-503 [127].

In the course of immunotherapy, PRMT5 has been linked to communication between immune and tumor cells. For example, in melanoma, PRMT5 represses the transcription of genes encoding nucleotide-binding oligomerization domain-like receptor family caspase recruitment domain containing 5 (NLRC5), inhibiting inflammation, and the combination of PRMT5 inhibition and immune checkpoint treatment limited the growth and enhanced the therapeutic effect of melanoma in mice [128]. Nevertheless, PRMT5’s role in cancer cells’ innate immune tolerance has received less attention. In the current study, the role of PRMT5 in controlling ferroptosis in TNBC’s immunotherapy response was investigated. It was discovered that PRMT5 can methylate and stabilize the ubiquitinated enzyme KEAP1, which inhibits the downstream NRF2/HMOX1 pathway and increases breast cancer resistance to ferroptosis and immunotherapy [129]. By controlling the transcription of the STAT1 gene through symmetric dimethylation of histone H3R2, PRMT5 knockdown in cervical cancer cells reduced PD-L1 expression and enhanced the quantity and activity of T lymphocytes inside the tumor microenvironment [130].

PRMT5 is a functionally complex and central cellular regulator that plays multi-faceted roles in cancer through epigenetic silencing, metabolic reprogramming, and signaling pathway regulation. However, its most noteworthy role lies in being a crucial bridge connecting the intrinsic oncogenic mechanisms of cancer cells with the tumor immune microenvironment.

**Table 2:** Role of protein arginine methyltransferases 5 in cancer pathogenesis, anti-tumor immunity, drug resistance, and cancer immunotherapy.

Cancer Type	Role(s) in Cancer	Mechanism of Action	Ref.
Breast cancer	Inhibition of ferroptosis of breast cancer cells	PRMT5→KEAP1/R596me2→KEAP1 ubiquitination (by TRIM25)↓→NRF2/HMOX1 expression↓→ ferroptosis of TNBC cells↓	[129]
	Attenuation of autophagy	PRMT5→methylation of ULK1→kinase activity and basal autophagic function↓	[131]
	Inhibition of cell proliferation	tamoxifen→PRMT5→methylation of Erα→transcription and cell proliferation↓	[132]
Cervical cancer	Promotion of cancer progression	PRMT5→H3R2me2s→transcription of STAT1↑→PD-L1 expression↑→development of cervical cancer↑	[130]
Chronic lymphocytic leukemia	Promotion of cancer progression	PRMT5→activates oncogenic signaling pathways→CLL development↑→Richter’s transformation	[133]
Colorectal cancer	Promotion of cancer cell dissemination	PRMT5→ methylation of SMAD4→activation of TGF-β signaling→promotion of CRC dissemination	[134]
	Promotion of cell proliferation, migration and invasion	PRMT5 interacts with MCM7→cell proliferation, migration, and invasion ↑	[135]
	cell proliferation	PRMT5→H3R8Me2s→activates LDHA→glycolysis↑→cell proliferation	[136]
	Promotion of CRC development	PRMT5 and EZH2→H3K27me3, H4R3me2s and H3R8me2s→CDKN2B (p15INK4b) expression↓→promotes CRC development	[137]
Glioma	Malignant phenotype	PRMT5→ERK1/2 pathway→malignant phenotype of glioma cells↑	[138]
	Promotion of self-renewal and proliferation	PRMT5→PTEN–AKT axis→GBM neurosphere self-renewal and proliferation↑	[139]
Hepatocellular carcinoma	Activation of de novo lipogenesis and tumorigenesis	PRMT5→methylation of SREBP1a→activates de novo lipogenesis and tumorigenesis	[140]
Liver cancer	Cell proliferation	PRMT5→ERK signaling→BTG2 expression↓→liver cancer cell proliferation	[141]
Lung cancer	Cell proliferation	PRMT5→methylation of KLF5→inhibit KLF5 degradation→promotes the maintenance and proliferation of lung cancer cells	[142]
	Growth inhibition	PRMT5 inhibition + anti-PD-L1→inhibits the growth of lung cancer cells and activates CD8+ T cell immune surveillance	[143]
	Cell proliferation and metastasis	PRMT5→activation of the FGFR3/Akt signaling axis→lung cancer cell proliferation and metastasis	[144]
Lymphoma	Cell survival	PRMT5→promotes WNT/β-CATENIN and AKT/GSK3β proliferative signaling→survival of lymphoma cells↑	[145]
Ovarian cancer	Tumor growth	PRMT5→methylation of ENO1→promotes active ENO1 dimer formation→glycolysis flux↑→tumor growth↑	[146]
Pancreatic cancer	Aerobic glycolysis and cell proliferation	PRMT5→expression of the tumor suppressor FBW7↓→stabilization of cMyc↑→aerobic glycolysis and pancreatic cancer cell proliferation↑	[125]
	Promotion of EMT	PRMT5→autophosphorylation of EGFR (Y1068 and Y1172)↑→activates Akt--β--catenin axis→promotes EMT	[147]
Prostate cancer	Promotion of cancer progression	circSPON2/miR-331-3p axis→PRMT5→H4R3me2s and H3R8me2s→CAMK2N1↓→PCa progression↑	[148]
	Cell growth	PRMT5 + Sp1 + Brg1→H4R3me2s→activates transcription of AR→prostate cancer cell growth↑	[149]

Arrows indicate direction of effect: (→) leads to/causes; (↑) increase/upregulation/activation; (↓) decrease/downregulation/inhibition.

### 3.2. PRMT9

Protein Arginine Methyltransferase 9 (PRMT9) was identified at the same time as PRMT8. Like PRMT7, PRMT9 contains two AdoMet-binding motifs. In addition, PRMT9 has two tetrapeptide repeats (TPRs) at its N-terminal end, which usually mediate protein–protein interactions [150]. At present, the biochemical characteristics of PRMT9 have not been determined, and few studies on PRMT9 have been reported. Among them, PRMT9 methylates the splicing factor SAP145 at the arginine 508 site and is functionally linked to the selective splicing regulation of precursor mRNAs [151]. PRMT9 also initiates the U2 snRNP that interacts with the structural domain of the motor neuron (SMN) [152].

Compared to other PRMTs, research on PRMT9 is at a very early stage. Its biochemical properties have not been fully determined, and very few substrates are known. PRMT9 can promote the development and progression of hepatocellular carcinoma and osteosarcoma. In hepatocellular carcinoma, PRMT9 promotes invasion and metastasis through activation of the PI3K/Akt/GSK-3β/Snail signaling pathway, and patients with high PRMT9 expression were found to have a short survival time and a high recurrence rate [153], indicating that PRMT9 is associated with poor tumor prognosis. In osteosarcoma studies, PRMT9 was found to act mainly through miRNA. MiRNA-543 inhibited PRMT9-enhanced cellular oxidative phosphorylation, and when miRNA-543 was absent, this promoted PRMT9 to increase HIF-1α instability and inhibit glycolysis in osteosarcoma cells [154], suggesting that miR-543/PRMT9/HIF-1α targeting the glycolytic pathway may be a potential therapeutic strategy to eradicate osteosarcoma cells. Methylation of arginine is essential for tumor maintenance. Acute myeloid leukemia is characterized by high levels of PRMT9, and leukemia is eradicated by inhibiting PRMT9 by reducing the arginine methylation of proteins involved in RNA translation and the DNA damage response. This sets off the cGAS–STING pathway, which in turn sets off leukemia-specific immune reactions. Targeting DNA damage response systems epigenetically may strengthen anti-tumor immunity [155]. These functionalities lack a unified main thread, exhibiting a high degree of mechanistic heterogeneity. In hepatocellular carcinoma and osteosarcoma, PRMT9 has been described as a factor promoting progression. However, inhibiting PRMT9 in AML can trigger an anti-leukemia immune response by activating the cGAS-STING pathway, suggesting that PRMT9 may play an immunosuppressive role in this context. This preliminary finding reveals that the function of PRMT9 may exhibit strong context dependency, similar to other PRMTs.

PRMT9, as a factor of significant interest in hepatocellular carcinoma (HCC) research, influences immune cell infiltration in the HCC immune microenvironment through various complex mechanisms. Promoting tumor cell invasion and metastasis: Overexpression of PRMT9 can enhance the invasive and metastatic capabilities of HCC. It is more frequently expressed in HCC tissues compared to adjacent non-cancerous tissues and is associated with malignant characteristics such as vascular invasion, tumor differentiation degree, and TNM staging. The alteration in the invasive and metastatic abilities of tumor cells can affect the structure and composition of the microenvironment, thereby influencing immune cell infiltration (e.g., by altering the extracellular matrix, remodeling the microenvironment at metastatic sites, and affecting the migration, recruitment, and distribution of immune cells). Regulation of epithelial–mesenchymal transition (EMT): PRMT9 modulates Snail expression by activating the PI3K/Akt/GSK-3β/Snail signaling pathway, thereby inducing the EMT and enhancing cell migration and invasion capabilities. The EMT alters the spectrum of cytokines, chemokines, and surface molecules secreted by tumor cells, consequently affecting the recruitment and mutual recognition of immune cells [153].

In most studies, type II PRMT enzymes are primarily known to act as transcriptional activators. However, certain biological processes reveal functional antagonism between type I and type II enzymes. For instance, PRMT6-mediated methylation of H3R2 suppresses transcription, while methylation of H4R3 promotes it. In contrast, PRMT5 exerts opposing effects: it activates transcription via H3R2 methylation and represses it through H4R3 methylation. Furthermore, PRMT5 and PRMT1 also play contrary roles in regulating cell viability—PRMT1 promotes apoptosis, whereas PRMT5 inhibits cell death. These observations suggest that symmetric dimethylarginine (SDMA) and asymmetric dimethylarginine (ADMA) may have complementary functions, potentially counteracting pathways that drive disease progression. Targeting these modifications could thus represent a novel therapeutic strategy for cancers involving aberrant PRMT expression.

## 4. Type III Protein Arginine Methyltransferase

PRMT7 functions as the only type III protein arginine methyltransferase in the current study, catalyzing the formation of MMA. Currently, the relationship between type III and type I and II enzymes is not understood, and there is no evidence that dimethylation of subsequent type I and II enzymes is triggered by PRMT7 monomethylation. The role and function of the type III enzyme PRMT7 are summarized below.

### PRMT7

Protein Arginine Methyltransferase 7 (PRMT7) is one of the PRMTs that contains two AdoMet-binding motifs. PRMT7 is a unique, evolutionarily conserved member of the PRMT family that catalyzes the monomethylation of arginine [156]. PRMT7 was identified late in the PRMT family. It is capable of symmetrically dimethylating histones H4R3 and H2AR3 (H4R3me2s, H2AR3me2s), which are linked to the transcriptional regulation of genes, as well as H3R2 (H3R2me2s). PRMT7 can methylate certain non-histone proteins, which are connected to the transcriptional activation of genes. PRMT7 regulates RNA-binding capacity and protein stability [157] and plays important functions in outer membrane development and innate immunity in zebrafish [158]. When DNA damage occurs, PRMT7 binds to BRG1 and modifies the DNA repair gene POLD1’s promoter using H4R3me2s and H2AR3me2s, which prevents this gene from being expressed. PRMT7 is also involved in the regulation of germ cell proliferation during the embryonic period [159] and plays a role in gene methylation in the male reproductive system by interacting with the CCCTC-binding factor CTCFL [160]. PRMT7 is expressed in male germ cells through miR- 877-3p/Col6a3, which downregulates sperm function in mice [161] (Table 3). PRMT7 may also play a role in the pluripotency of embryonic stem cells (ESCs), as its expression is lost during ESC differentiation [162]. Another study noted that PRMT7-deficient mouse embryonic fibroblasts (MEFs) exhibit premature senescence, with increased expression of the cell cycle inhibitors p16 and p21 [163]. In contrast, humans deficient in PRMT7 are characterized by significant intellectual disability, hypotonia, and facial dysmorphism [164]. The anti-aging effect of PRMT7 is not only found in fibroblasts; it also affects the aging of muscle stem cells. In skeletal muscle, PRMT7 acts as a regulator of the DNMT3b/p21 pathway to maintain the regenerative capacity of static muscle stem cells (satellite cells), and when PRMT7 is absent, the same cell cycle block and premature cellular senescence occur [165], possibly due to the impairment of satellite cell regeneration and self-renewal capacity. The immune system’s reaction to PRMT7 deletion may include a decrease in mature marginal B cells and an increase in tiny follicular B cells, which will aid in the development of germinal centers following immunization. To treat Bcl6 with H4R3me1 and H4R3me2s, PRMT7 is recruited to the promoter of the protein, hence preventing this gene’s expression. PRMT7 deficiency also results in decreased oxidative metabolism and age-related obesity [166]. PRMT7 primarily inhibits adipogenesis by regulating C/EBP- activity [167] and methylates p38MAPK to promote myogenic cell differentiation [168], which implies that PRMT7 is a novel regulator of muscle oxidative metabolism.

An essential component of cell carcinogenesis is the relationship of the post-translational methylation of PRMT and its proteins with tumor development and growth. Additionally, PRMT7 has been shown to have a major regulatory function in a range of cancer cells. Through its regulation of the -catenin/C-MYC pathway [169], PRMT7 stimulates the formation of renal cell carcinoma [169]. When it comes to human non-small-cell lung cancer, it also facilitates metastasis through its interactions with HSPA5 and EEF2 [170]. Based on current research, PRMT7 mostly methylates arginine and glycine moieties, according to its substrate range and relationship with type I and type II PRMT substrates. Among these, methylation is abundant in proteins connected to splicosome- and RNA-related pathways, and many of the arginine methylation sites controlled by PRMT7 are close to the phosphorylation sites. These methylation sites and proximal sequences are susceptible to cancer mutations. PRMT7 is increased in prostate, colorectal, and breast cancer cells, leading to aberrant alternative splicing and elevated hnRNPA1 arginine methylation [171]. The progression of breast cancer is primarily linked to PRMT7. PRMT7-mediated EMT metastasis is promoted by H4R3 histone methylation changes, which decrease E-cadherin expression in breast cancer cells. PRMT7 has been shown by Yao et al. to co-precipitate with transcription factors YY1 and HDAC3 to decrease E-cadherin accumulation, which in turn promotes tumor cell metastasis. Cancer cells can infiltrate tissues because matrix metalloproteinases (MMPs) break down the extracellular matrix. Relevant research has demonstrated that basal breast cancer cells overexpress PRMT7, which increases the production of MMP2 and MMP9 to encourage cancer cell invasion and metastasis. Conversely, blocking PRMT7 expression can lessen cancer cell migration and invasion. According to research conducted by Geng’s team, PRMT7 in breast cancer is associated with both epigenetic modifications in the methylation modification of its substrate and self-methylation processes that are closely linked to the metastasis of breast cancer. Furthermore, self-methylation modification at particular sites is crucial for the clinical diagnosis and management of breast cancer [172].

Like PRMT3 and PRMT6, PRMT7 also plays a role in antiviral therapy [173], and whether there is an interaction between them in this process needs to be verified by numerous experiments. It has been found that PRMT7 regulates the expression of RUNX1 target genes in T cell acute lymphoblastic leukemia (T-ALL) and plays an active role in the pathogenesis of T-ALL [174]. As a crucial member of the protein arginine methyltransferase family, PRMT7 plays multifaceted and pivotal roles in the immune microenvironment. PRMT7 is closely associated with tumor immune cell infiltration; in melanoma, inhibition of PRMT7 increases immune cell infiltration, triggering anti-tumor T cell responses to suppress tumor growth. TCGA data indicate that its expression is negatively correlated with T cell infiltration, potentially serving as a key factor in melanoma immune evasion; in PRMT7-knockout B16.F10 melanoma, the expression of genes related to the interferon pathway, antigen presentation, and chemokine signaling is upregulated, facilitating the recruitment and activation of immune cells. PRMT7 also influences the tumor immune microenvironment by regulating immune checkpoint molecules: in melanoma cells, it acts as a coactivator of IRF-1, promoting PD-L1 expression through upregulation of the promoter H4R3me2s; the binding of PD-L1 to PD-1 on T cells inhibits T cell activity, aiding tumor immune evasion, while inhibiting PRMT7 may disrupt this evasion and enhance the efficacy of immunotherapy [175].

**Table 3:** 5 Phenotypic outcomes of homozygous and heterozygous, conditional and full-body knockout in mice models for PRMTs.

PRMT Isoform	Knockout Type	Model Description	Viability/Lethality	Key Tissue/Cellular Phenotypes	Ref.
PRMT1	Conventional Homozygous KO	Gene trap	Embryotoxicity		[16]
	Conditional Homozygous KO	ERT2-Cre; endothelial cell-specific knockout	Viability	Worsening of pulmonary hemorrhage	[176]
	Conditional Homozygous KO	Mx1-Cre; adult hematopoietic cell-specific knockout	About one in eight mice died within five months	Prominent effect on adult hematopoiesis	[177]
	Conditional Homozygous KO	Ngn3-Cre; germ cell-specific knockout	Viability	Prmt1 KO male mice were completely infertile; female KO mice were fertile	[178]
	Conditional Homozygous KO	Vil-CreERT2; intestinal epithelium-specific knockout	Viability	Intestinal cell proliferation and crypt elongation increased in mice aged 8–12 weeks	[179]
	Conditional Homozygous KO	Myh6-cre; myocardium-specific knockout	Mouse lethality began at four weeks of age and nearly all died within two months		[180]
	Conditional Heterozygous KO	Myh6-cre; myocardium-specific knockout	25 percent of mice were dead within two months		[180]
	Conditional Heterozygous KO	Myl1-Cre; skeletal muscle-specific knockout	Viability	Muscle loss	[181]
	Conventional Homozygous KO	Cre/loxP recombination system	Embryos did not survive to 7.5 days		[17]
	Conditional Homozygous KO	Cre/loxP recombination system; epidermis-specific knockout	Small body size, thin clinical epidermis, and perinatal mortality		[182]
	Conditional Homozygous KO	Flp-Cre; hepatocyte-specific knockout	Viability		[183]
PRMT2	Conventional Homozygous KO	Standard gene targeting	Viability	There is no obvious difference in the display form	[45]
PRMT3	Conventional Homozygous KO	Standard gene targeting	Viability; mouse embryos targeted to disrupt PRMT3 were smaller but survived after birth and reached normal mouse size as adults		[57]
	Conditional Homozygous KO	Alb-Cre; liver-specific knockout	Viability	Inhibited tumor progression and increased CD8+ T cell infiltration in mouse tumors	[184]
CARM1	Conventional Homozygous KO	Standard gene targeting	Mouse embryos survived to birth and responded to stimuli but developed respiratory distress and died within 20 min		[185]
PRMT5	Conditional Homozygous KO	MyoDCre; myogenic lineage-specific knockout	Adult mice died prematurely	Muscle atrophy and early death	[186]
	Conditional Homozygous KO	Cre-loxP; medullary thymic epithelial cell-specific knockout	Viability	Smaller thymus in mice	[187]
	Conditional Homozygous KO	Myl1-CRE; skeletal muscle-specific knockout	Viability	Muscle mass, oxidative capacity, power generation, and exercise performance decreased in mice	[188]
	Conditional Homozygous KO	Pdx 1-CreER; islet-specific knockout	Viability	Decreased glucose tolerance in mice	[189]
	Conditional Homozygous KO	OC-Cre; osteoblast-specific knockout	Viability	Delayed socket healing in Prmt5 knockout mice	[190]
	Conventional Homozygous KO	Mx1-Cre	Two weeks after the first induction, the mice developed severe anemic pallor, and most of the mice with PRMT5 deficiency died within 1–2 days	Deletion of PRMT5 during adult hematopoiesis leads to severe cytopenias	[191]
PRMT6	Conventional Homozygous KO	CRISPR-Cas9	Viability	Enhanced the body’s innate antiviral immunity	[87]
	Conventional Homozygous KO	EIIa-Cre	Viability		[192]
PRMT7	Conditional Homozygous KO	Cdh5-CreERT2; endothelial cell-specific knockout	Viability	Increased apoptosis and fibrosis impair heart recovery	[193]
	Conventional Homozygous KO	Standard gene targeting	Viability	Muscles show a shift to glycolytic fiber types	[166]
	Conditional Homozygous KO	Myh6-Cre; cardiac-specific knockout	Viability; birth rate decreased by about half	Plays a protective role in Ang II-induced cardiomyopathy	[194]
PRMT8	Conventional Homozygous KO	Ayu1-Cre	Viability	Acetylcholine and choline decreased while PC levels increased	[95]
	Conventional Homozygous KO	Standard gene targeting	Viability	In mice, the composition of phospholipids was changed, mitochondrial stress capacity was reduced, and neuroinflammatory markers were increased	[98]
PRMT9	Conventional Homozygous KO	CMV-Cre	Some died after birth	Sea horse neurons showed impaired excitatory synapse development	[195]

## 5. Conclusions

Protein arginine methylation is a PTM involved in many key cellular pathways that regulate cellular physiological activities and fate decisions and is aberrantly expressed in various types of human cancers. Expression of PRMTs and arginine demethylases and altered enzymatic activity are involved in the development of a variety of malignancies, and PRMTs are generally overexpressed in tumors. Based on the available studies, it is evident that the members of PRMTs do not act independently, and they often interact with each other in terms of biological function and jointly regulate a certain physiological role. Therefore, it is necessary to observe the changes in other enzymes while studying one enzyme in the disease to better understand the mechanism of action of the disease’s occurrence and to find key therapeutic approaches. Although much has been reported on PRMTs, there are still many shortcomings that need to be further explored. For example, there is a lack of effective specific inhibitors of PRMTs, and although several PRMT inhibitors have been successfully constructed, their selectivity and specificity for different PRMTs need to be improved. Furthermore, additional experiments are needed to determine the efficacy of these drugs in vivo. Moreover, nine PRMTs have been shown to catalyze modifications of ADMA and SDMA, and additional arginine demethylases likely remain to be identified. PRMT1, PRMT3, PRMT5, PRMT6, PRMT7, and PRMT8 preferentially methylate histone H4, whereas PRMT4 preferentially methylates histone H3. Some studies have found that the substrate environment has a dramatic effect on the histone arginine methylation activity of PRMTs, but the current understanding of the molecular mechanisms underlying PRMT substrate recognition is incomplete 107.

Features of the tumor microenvironment can be broadly classified into three groups: hypoxia, chronic inflammation, and immunosuppression. As a result of their complementary roles in the formation of the complex mechanism network that is crucial to the growth of tumors, the relationship between PRMTs and the tumor microenvironment is becoming increasingly clear. (a) Hypoxia: All solid tumors experience hypoxia, which often starts at the tumor’s center and progressively diminishes in degree. Research has demonstrated that hypoxia-inducing factor (HIF) signaling pathways play a major role in the biological reactions that follow intratumoral hypoxia. For example, PRMT5 is expected to stimulate tumor growth by inducing cell proliferation and facilitating the activation of HIF-1 to help establish a microenvironment that is friendly to the tumor. (b) Prolonged inflammation: Inflammation stimulates the growth and invasion of cancer cells, the remodeling and breakdown of the matrix, the formation of new blood vessels, and the suppression of the immune system. The microenvironment contains local signaling molecules that can replenish and activate M2 (IL-10HIL-12L) macrophages, which are primarily engaged in chronic inflammation and Th2 cell production. Examples of these molecules include prostaglandin E2 (PGE2), IL-4, IL-6, IL-10, TGF-β, and M-CSF; they encourage the growth and incidence of malignancies. For instance, abnormal inflammatory signaling in PRMT2-depleted AML cell lines has been linked to an excess of IL-6 generated as a consequence of dysregulated NF-κB signaling, which in turn results in an overactivation of STAT3. (c) Immunosuppression: The microenvironment is a “black hole region” for the body’s anti-tumor immune response and immune intervention, making it difficult to kill tumor cells. These abnormalities include cytotoxic T lymphocyte-associated antigen 4 (CTLA-4), PD-1/PD-L1, immunosuppressive Treg cells, MDSC, intratumoral blood vessels, and indoleamine 2, 3-dioxygenase (IDO), among others. One way to reduce the expression of PD-L1 and increase the quantity and activity of T lymphocytes in the tumor microenvironment is to knock down PRMT5 in cervical cancer cells. This can be achieved by controlling the transcription of the STAT1 gene. Increasing evidence has shown that PRMTs are involved in the immune response. Therefore, manipulating PRMTs could be a feasible strategy to modulate tumor microenvironment for immunotherapy.

In addition to physiological analysis, this area of research requires additional molecular studies to better understand how and why arginine methylation is dysregulated in cancer. Therefore, exploring the pathogenic mechanisms of PRMTs for diseases, especially cancer, and selecting specific PRMTs as targets for drug intervention has promising applications for the treatment of diseases.

## Data Availability

Not applicable.
